# Editorial: Metabolic changes in vascular disorders: A path for early diagnosis and new therapeutic targets

**DOI:** 10.3389/fcvm.2023.1163298

**Published:** 2023-03-03

**Authors:** Sana Ayyoub, Isabel Blanco, José Luis Izquierdo-García, Olga Tura-Ceide

**Affiliations:** ^1^ Pneumology Department, Girona Biomedical Research Institute - IDIBGI, Girona, Spain; ^2^ Department of Pulmonary Medicine, Hospital Clínic-Institut d’Investigacions Biomèdiques August Pi i Sunyer (IDIBAPS), University of Barcelona, Barcelona, Spain; ^3^ Pneumology Department, Centro de Investigación Biomédica en Red de Enfermedades Respiratorias (CIBERES), Madrid, Spain; ^4^ Biomedical Imaging Group, Instituto Pluridisciplinar, Complutense University of Madrid, Madrid, Spain; ^5^ Department of Chemistry in Pharmaceutical Sciences, Pharmacy School, Complutense University of Madrid, Madrid, Spain

**Keywords:** metabolism, diagnosis, cardiopulmonary diseases, endothelial dysfunction, therapeutic targets

**Editorial on the Research Topic** Metabolic changes in vascular disorders: A path for early diagnosis and new therapeutic targets

Abnormal metabolic pathways have been associated with different pathological conditions, including disorders of the vascular system. Vascular disorders include atherosclerosis, hypertension, deep vein thrombosis, and pulmonary embolism, which all involve abnormalities of the vessels. This Editorial combines information from seven recent reports under the Research Topic “Metabolic Alterations in Cardiopulmonary Vascular Dysfunction” and aims to shift attention toward the targeting of metabolic alternations as a possible therapeutic approach for the management of vascular disorders and/or establishing them as new markers for early diagnosis.

Vascular disorders encompass different types of pathological conditions, and so far, there have been no reports on the prevalence of all types of vascular disorders combined. However, cardiovascular disorders have been reported to be a leading cause of death worldwide, affecting approximately 523 million people (1) and accounting for 32% of total deaths in 2019 according to the World Health Organization.

The targeting of metabolic pathways has emerged as a possible therapeutic strategy for cancer management (2). Considering that vascular disorders and cancer share some similar metabolic alternations, this strategy can also be employed in the management of vascular disorders, supported by the findings from the reports included in this collection.

In a review by Liu et al. pulmonary hypertension presents with hyperproliferative vascular cells and shares a similar metabolic pathway with cancer in terms of activated aerobic glycolysis promoting cellular proliferation. Two other altered metabolic pathways are also reported, including glutamine metabolism and the Randle cycle. The authors encourage further evaluation of these pathways as possible therapeutic targets for the management of pulmonary hypertension as the currently available drugs do not halt disease progression nor do they reverse pulmonary vascular remodeling (Liu et al.).

By performing targeted metabolomics and multi-omics in rat models of pulmonary hypertension-induced right ventricular failure (PH-RVF), several common metabolites from key pathways, such as glycolysis, fatty acid metabolism, oxidative phosphorylation, and the tricarboxylic acid (TCA) cycle, were found to be significantly altered, highlighting the importance of considering these as targets for PH-RVF treatment (3).

In another review by Liu et al. it was reported that vascular calcification, a hallmark of cardiovascular disease, is attributed to altered metabolic pathways contributing to mitochondrial dysfunction. This in turn can promote oxidative stress and alter calcium homeostasis. Thus, based on the compelling evidence of the role of abnormal mitochondrial metabolic pathways in vascular calcification, it is important to consider this organelle and its metabolic pathways as a therapeutic target for vascular calcification (Liu et al.).

Genetic variations can also contribute to alterations in the metabolic pathways, and the response to drugs can be different among individuals. One review highlighted the importance of the genotype-guided use of purinergic receptor type Y subtype 12 (P2Y12) inhibitors as an anti-platelet therapy for preventing bleeding and thrombotic complications (Al-Abcha et al.). Considering the importance of the genetic testing guided therapy, promoting their implementation among the health care systems is highly encouraged, especially with narrow therapeutic window drugs.

Studying the causative factors for threatening illnesses is also important for targeting and managing these factors to prevent them from contributing to more serious conditions. A study that used two-sample Mendelian randomization (TSMR) analysis from the available genome-wide association studies (GWASs) has found that blood pressure (BP) is a strong causal effect of myocardial infarction (MI) and there is no causal effect of MI on BP, thus recommending the optimization of BP control to prevent MI, and suggesting that hypertension medications can be used to effectively prevent MI (Yang et al.).

Lastly, it is also important to consider these metabolic changes as possible biomarkers for early diagnosis to halt disease progression. One study analyzed gene expression data from the Gene Expression Omnibus database and identified six metabolism-related genes as potential key players in the development of dilated cardiomyopathy (DCM). They reported that the oxoglutarate dehydrogenase L (*OGDHL*) gene, which is a metabolism-related gene, is upregulated in DCM patients along with a higher OGDHL protein level; thus, it can be considered as a potential biomarker for myocardial remodeling in DCM (Tang et al.). Also, D-dimer, which is a product of fibrin degradation, along with electrocardiogram (ECG) abnormality, have been found to be significantly associated with illness severity and death in patients infected with severe acute respiratory syndrome coronavirus 2 (SARS-CoV-2), probably related to SARS-CoV-2-induced thrombosis; thus, they can be employed as biomarkers for predicting disease severity, and patients with higher D-dimer levels can be monitored early with more care provided throughout their illness (Chen et al.).

This collection gathers information from compelling reports on the role of metabolic abnormalities in vascular disorders, highlighting the importance of considering them as possible therapeutic targets and early diagnostic markers for the management of the different pathological conditions associated with vascular abnormalities.
